# Realizing a deterministic source of multipartite-entangled photonic qubits

**DOI:** 10.1038/s41467-020-18635-x

**Published:** 2020-09-28

**Authors:** Jean-Claude Besse, Kevin Reuer, Michele C. Collodo, Arne Wulff, Lucien Wernli, Adrian Copetudo, Daniel Malz, Paul Magnard, Abdulkadir Akin, Mihai Gabureac, Graham J. Norris, J. Ignacio Cirac, Andreas Wallraff, Christopher Eichler

**Affiliations:** 1 Department of Physics, ETH Zurich, Zurich, CH-8093 Switzerland; 2 Max-Planck-Institute of Quantum Optics, Hans-Kopfermann-Strasse 1, Garching, 85748 Germany; 3 Munich Center for Quantum Science and Technology, Schellingstrasse 4, München, 80799 Germany; 4 Quantum Center, ETH Zurich, Zurich, CH-8093 Switzerland

**Keywords:** Qubits, Single photons and quantum effects

## Abstract

Sources of entangled electromagnetic radiation are a cornerstone in quantum information processing and offer unique opportunities for the study of quantum many-body physics in a controlled experimental setting. Generation of multi-mode entangled states of radiation with a large entanglement length, that is neither probabilistic nor restricted to generate specific types of states, remains challenging. Here, we demonstrate the fully deterministic generation of purely photonic entangled states such as the cluster, GHZ, and W state by sequentially emitting microwave photons from a controlled auxiliary system into a waveguide. We tomographically reconstruct the entire quantum many-body state for up to *N* = 4 photonic modes and infer the quantum state for even larger *N* from process tomography. We estimate that localizable entanglement persists over a distance of approximately ten photonic qubits.

## Abstract (web summary)

Deterministic generation of photonic multi-partite entangled states has previously been achieved for specific states using ad-hoc devices. Here, the authors present a single superconducting circuit device to deterministically generate a variety of states, namely W, GHZ, and cluster states.

## Introduction

Entanglement is one of the most fundamental concepts in quantum physics^1^ and an essential resource for applications in quantum information processing^2,3^. Both the theory of entanglement^4^ and the experimental generation of entangled states of light^5–10^ and matter^11–13^ have therefore been a subject of intense research. Of particular importance are multipartite entangled states of photons for their use in quantum communication and network protocols^14^. Experiments to generate entangled states of light most commonly rely on spontaneous parametric down-conversion sources and heralding^5–7^. The probabilistic nature of such schemes is a major obstacle when scaling to larger systems, which has motivated the study of deterministic sources of entangled photonic states more recently^8–10^. So far, only states within device-specific classes of entanglement have been generated deterministically and with a moderate size as compared to their matter-based counterparts^11,13^. Achieving more versatility in the generation of entanglement has therefore been an outstanding challenge, which we address in this work.

**Fig. 1:**
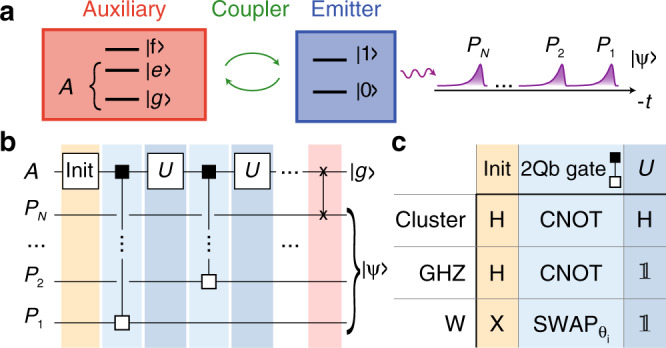
Generation of entangled states of photons. **a** An auxiliary quantum system *A* coupled to an emitter is used to generate a state $\left|\psi \right\rangle$ of photonic modes *P*_*i*_. **b** Quantum circuit using an initialization gate and single-qubit gates *U* acting on the auxiliary system *A*, and two-qubit gates (small squares) of the CNOT and SWAP family between the auxiliary qubit and the photons *P*_*i*_. The last two-qubit gate is always a SWAP (crosses). **c** Specific gates used for the generation of the cluster state, the GHZ state, and the W state. H: Hadamard gate, 1: Identity gate.

A generic protocol to generate entangled states as a train of sequentially emitted photons was proposed by Schön et al.^15^ and is based on a long-lived auxiliary quantum system *A*, which sequentially interacts with an emitter qubit via a controllable coupling (see Fig. 1a). After each interaction cycle *i*, the engineered emission into a waveguide converts the state of the emitter qubit into a flying photonic qubit *P*_*i*_ defined by the presence or absence of a single photon in the associated time bin. The combination of controllable interaction and photon emission can be understood as a two-qubit gate realized between *A* and *P*_*i*_. Consequently, the photonic state preparation is formally described by repeated unitary operations *U* applied to *A* interleaved with two-qubit gates, as represented by the generic quantum circuit shown in Fig. 1b. The range of accessible states crucially depends on the set of available two-qubit gates. Although the GHZ state and the cluster state require a controlled NOT (CNOT) gate only^16^ and have already been generated experimentally^8^, the preparation of a W state relies on the ability to perform SWAP(*θ*_*i*_) gates with adjustable rotation angle ${\theta }_{i}=2\arcsin \left[{(N-i+1)}^{-0.5}\right]$ (see Fig. 1c). Furthermore, a final SWAP operation is required to disentangle the photonic qubits from the auxiliary system by bringing the latter back to its ground state $\left|g\right\rangle$, thereby rendering the generation of the final photonic state $\left|\psi \right\rangle$ fully deterministic.

In this work, we realize a superconducting circuit-based source of entangled microwave photons and implement the above protocol with both generic SWAP- and CNOT-type gates to demonstrate deterministic state preparation of cluster, GHZ, and W states. We perform full quantum tomography of states up to four modes and use process tomography to analyze the localizable entanglement in larger photonic states. We infer than entanglement persists over a distance of approximately ten photonic qubits.

## Results

### Superconducting circuit implementation

**Fig. 2:**
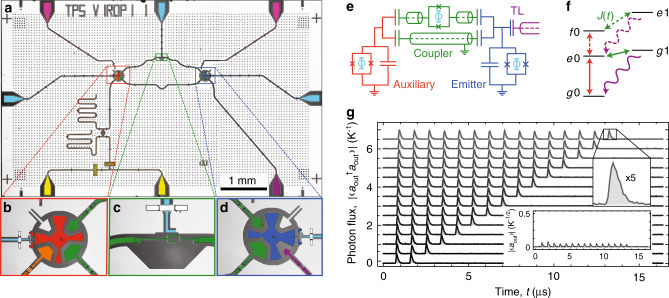
Experimental implementation with a superconducting circuit. **a** False color optical micrograph of the chip used in the experiment, comprising an auxiliary qutrit (red, **b**), a tunable coupler (green, **c**), an emitter qubit (blue, **d**), an output transmission line (purple) connecting to the measurement chain, a feedline (yellow), a readout circuit with Purcell filter (orange), three flux lines (cyan), and two charge lines (pink). **e** Equivalent electrical circuit diagram. The auxiliary and the emitter qubits are transmons, with a flux tunable superconducting quantum interference device (SQUID) loop capacitively shunted to ground. The coupler consists of two paths, one mainly inductive (top), the other capacitive (bottom). Photons are emitted into the semi-infinite transmission line (TL). **f** Energy level diagram, indicating the possible transitions induced by single-qubit gates (red), parametric modulation of the coupler (green), and photon emission via spontaneous decay into the TL (purple). **g** Measured outgoing photon flux $\left|\langle {a}_{{\rm{out}}}^{\dagger }{a}_{{\rm{out}}}\rangle \right|$ vs. time in the transmission line, in units of inverse emitter linewidth *κ*, for cluster states of *N* = 2, . . . , 15 modes. Traces are offset by 0.5 for clarity. Insets: zoom-in by a factor 5 of the last time bin for clarity, and amplitude $\left|\langle {a}_{{\rm{out}}}\rangle \right|$ in units of *κ*^−1/2^, for the cluster state of 15 modes.

We realize the auxiliary system as a transmon (see Fig. 2, red) of which we use the first three energy level $\left|g\right\rangle$, $\left|e\right\rangle$, and $\left|f\right\rangle$ with transition frequencies *ω*_*ge*_/2*π* = 5.758 GHz and *ω*_*ef*_/2*π* = 5.455 GHz (see Supplementary Notes 1 and 2 for details about the device fabrication, the experimental setup, and sample characterization). We perform local unitary operations between the states of the auxiliary qutrit system by applying microwave pulses with controlled amplitude and phase resonant with *ω*_*ge*_ and *ω*_*ef*_, respectively (red arrows in Fig. 2f). To stimulate state-dependent photon emission processes, we tunably couple the auxiliary transmon to a second transmon (blue), acting as the emitter, which is operated at *ω*_01_/2*π* = 5.896 GHz and decays into a semi-infinite transmission line with rate *κ*/2*π* = 1.95 MHz. The coupling between the auxiliary system and the emitter is mediated by two parallel channels, one of which is tunable via the magnetic flux applied to a superconducting quantum interference device loop. This specific coupler arrangement (green) allows us to interferometrically cancel the static coupling and protect the auxiliary transmon from Purcell decay into the transmission line while enabling fast decay of the emitter^17,18^.

By parametrically modulating the applied flux around the value at which the static coupling is canceled, we selectively drive sideband transitions between excitation-number-conserving states (green arrows in Fig. 2f) with a rate *J*_ac_/2*π* ≃ 5 MHz. To perform SWAP(*θ*_*i*_) gates between the auxiliary qubit and the emitter qubit, we modulate the flux at the difference frequency Δ_e0g1_/2*π* = (*ω*_01_ − *ω*_*ge*_)/2*π* = 139 MHz driving a transition between the states $\left|e0\right\rangle$ and $\left|g1\right\rangle$. The CNOT gate is a photon emission process conditioned on the auxiliary qubit being in its first-excited state. We realize this state-dependent photon generation by first applying a *R*_y_(*π*) pulse on the *e*–*f* transition of the auxiliary qubit and then driving a sideband transition at frequency Δ_f0e1_/2*π* = (*ω*_01_ − *ω*_*ef*_)/2*π* = 441 MHz between the $\left|f0\right\rangle$ and $\left|e1\right\rangle$ state. This sequence leaves the auxiliary qubit in the $\left|e\right\rangle$ state while emitting a photon.

By controlling amplitude, duration, and phase of the pulses, we choose any targeted SWAP and CNOT angle^19^ (see Supplementary Note 3 for details). Using a continuous set of two-qubit gates^20^ allows for the generation of a family of entangled states belonging to the class of matrix-product states (MPS) with bond dimension *d* = 2^21^ of which specific instances are studied in this work.

### State generation by sequential emission

By applying sequences of the described gates, according to the quantum circuit shown in Fig. 1b, c, we generate many-body states of microwave radiation and experimentally assess their properties through heterodyne measurements of the output field *a*_out_ in the transmission line (see Supplementary Note 4 for information about the detection method). We first measure the temporal profile of the pulse train by averaging the photon flux $\left|\langle {a}_{{\rm{out}}}^{\dagger }{a}_{{\rm{out}}}\rangle \right|$ over 10^6^ repetitions of the experiment (see Fig. 2g) for the example of prepared cluster states with up to 15 photonic qubits. Each pulse has an initial rise time of *~π*/*J*_ac_ ≃ 100 ns corresponding to the duration of the two-qubit gate, before exponentially decaying with characteristic timescale *κ*^−1^ ≃ 80 ns given by the decay rate of the emitter into the transmission line (see inset). We have chosen a conservative repetition time of *T* = 900 ns, to ensure that the emitter has fully returned into its ground state before starting the next emission cycle. Each time bin contains an integrated photon flux of ideally half a photon for the cluster state. In comparison to the photon flux, the field expectation value $\left|\langle {a}_{{\rm{out}}}\rangle \right|$, shown in the inset, is close to zero, as expected for the cluster state. We attribute the small but finite measured $\left|\langle {a}_{{\rm{out}}}\rangle \right|$ to coherent errors at the 1% level in single-qubit *e*–*f* pulses.

### Full tomography of states up to four modes

**Fig. 3:**
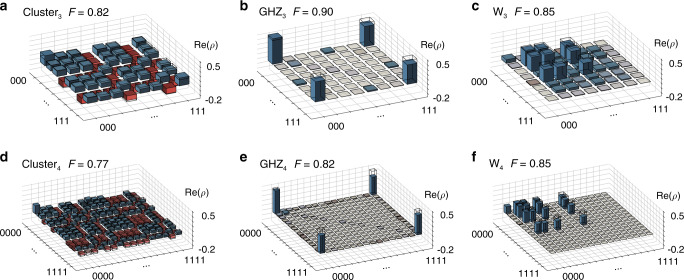
Complete state tomography. **a**–**c** Three- and **d**–**f** four-mode state tomography. We plot the real part ${\rm{Re}}(\rho )$ of the most likely density matrices *ρ* (bars) for **a**, **d** the cluster state, **b**, **e** the GHZ state, and **c**, **f** the W state. Absolute values of the imaginary part (not shown) are all below 0.03. Ideal density matrices *ρ*_ideal_ are shown as black wireframes. The fidelity $F={\rm{Tr}}{\left(\sqrt{\sqrt{\rho }{\rho }_{{\rm{ideal}}}\sqrt{\rho }}\right)}^{2}$ is indicated. Entries are following the binary ordering from (0)000 to (1)111.

To extract the quantum-mechanical properties of the generated many-body states, we perform quantum state tomography. We reconstruct the density matrix for up to four modes, enabled by advancing the capabilities of our field programmable gate array (FPGA)-based data acquisition (see Fig. 3 and Supplementary Note 4 for a detailed discussion of the tomography method). Reconstructing the joint density matrix of four modes is well beyond what has been demonstrated previously for propagating microwave fields, which is full tomography for up to two modes^22–24^. We note that full tomography for even more than four modes is possible by further extending the capacity of data storage on the FPGA or by using offline data processing solutions.

We find that the cluster state contains non-vanishing values in all entries of the density matrix, with magnitude close to 2^−*N*^, indicating that all basis states are occupied with nearly equal probability. The characteristic pattern of sign changes in some individual terms, which cannot be produced by local *Z* gates, as there is an imbalance between the number of basis states with positive and negative sign, and renders this state inseparable. For the GHZ state, defined as the equal superposition of the absence or presence of a single photon in each mode ${{\rm{GHZ}}}_{N}=\left({\left|0\right\rangle }^{\otimes N}+{\left|1\right\rangle }^{\otimes N}\right)/\sqrt{2}$, only the four corners of the density matrix have a non-zero value, equal to 0.5. Finally, the W state ${{\rm{W}}}_{N}=\mathop{\sum }\nolimits_{i = 1}^{N}{\left|0\right\rangle }^{\otimes (i-1)}\left|{1}_{i}\right\rangle {\left|0\right\rangle }^{\otimes (N-i)}/\sqrt{N}$ is restricted to entries containing exactly one excitation. All non-vanishing entries have ideally the same value of *N*^−1^. In all six cases, the experimentally reconstructed density matrices are in very good agreement with the ideally expected ones, which is indicated by the high fidelities *F*_Cluster_ = 0.82 (0.77), *F*_GHZ_ = 0.90 (0.82), and *F*_W_ = 0.85 (0.85) for *N* = 3 (4), with $F={\rm{Tr}}{\left(\sqrt{\sqrt{\rho }{\rho }_{{\rm{ideal}}}\sqrt{\rho }}\right)}^{2}$, compared to the respective ideal state *ρ*_ideal_. For nearly all prepared states, we observe a higher probability to be in the ground state compared to the ideal case and an overall decrease in the population of states with photons emitted in late time bins. We attribute these effects to the relaxation of the auxiliary qubit during the sequential emission process. Indeed, master equation simulations, taking finite relaxation times ${T}_{1}^{(e)}=21\,$μs (${T}_{1}^{(f)}=7\,$μs) and Ramsey dephasing times ${T}_{2}^{\star (e)}=17\,$μs (${T}_{2}^{\star (f)}=8\,$μs) into account, indicate that the majority of the infidelity originates from the finite coherence of the auxiliary qubit. For the four-mode states, e.g., we simulate fidelities ${F}_{{\rm{Cluster}}}^{{\rm{(MES)}}}=0.87$, ${F}_{{\rm{GHZ}}}^{{\rm{(MES)}}}=0.89$, and ${F}_{{\rm{W}}}^{{\rm{(MES)}}}=0.90$ to the respective ideal state. In general, the W state is affected by decoherence of the first-excited state only and thus pertains a higher fidelity at intermediate mode number *N* but requires the experimental calibration of more gates. The GHZ state has a higher simulated fidelity at very large mode number *N* > 10 due to its finite overlap of 0.5 with the vacuum state, whereas the overlap with vacuum scales as 1/*N* for the cluster state and is 0 for the W state.

### Localizable entanglement

**Fig. 4:**
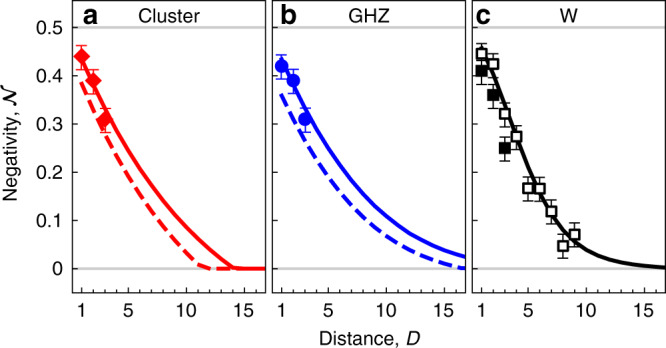
Localizable entanglement. Negativity ${\mathcal{N}}$ between the first and (*D* + 1)-th photonic mode separated by a distance *D*. Filled dots indicate measurements based on complete tomography, dashed lines are inferred from process maps, empty dots are calculated from partial tomography (see text), and solid lines indicate the coherence limit from master equation simulations. Ideal value of ${\mathcal{N}}$ is 0.5. The cluster state (**a**, red diamond), GHZ state (**b**, blue circles), and W state (**c**, black squares) data are shown. Error bars represent the statistical SD of 0.025 estimated via repeated nominally identical measurements.

To quantify entanglement in the generated many-body states of light, we analyze the localizable entanglement between the first and the last qubit in the chain^25^ after projecting out all other qubits to obtain the two-qubit density matrix *ρ*_*D*_ as a function of their distance *D* = *N* − 1. As a metric, we choose to report the negativity ${\mathcal{N}}({\rho }_{D})={\sum }_{i}\left|{\lambda }_{i}\right|$, where *λ*_*i*_ are the negative eigenvalues of the partial transpose of *ρ*_*D*_, which is an entanglement monotone for bipartite systems and hence a suited measure of entanglement. For the data points obtained from full tomography (see Fig. 4, filled markers), *ρ*_*D*_ is calculated from the full density matrix by projecting all qubits but the first and last into their respective ground states in the *X*- (*Z*-)basis for the cluster and GHZ (W) states. (The original definition of localizable entanglement averages over all possible outcomes in a given measurement basis. For the W state in particular, localizable entanglement only persists in case the measurement outcome is the ground state for all measured qubits. The resulting estimate for the entanglement length is independent of this particular choice.) The resulting negativities ${{\mathcal{N}}}_{{\rm{Cluster}}}=0.44,0.39,0.31$, ${{\mathcal{N}}}_{{\rm{GHZ}}}=0.42,0.39,0.31$, and ${{\mathcal{N}}}_{{\rm{W}}}=0.41,0.36,0.25$ decrease monotonically with *N* = 2, 3, 4 (*D* = 1, 2, 3) as expected and in good agreement with master equation simulations (solid lines) taking the finite coherence of the auxiliary system into account. The deviation between results from full tomography (solid symbols) and partial tomography (open symbols) is likely due to small parameter drifts accumulated during the time in between taking the two different data sets.

Although tomography of the most general *N*-qubit state requires exponentially growing resources for data acquisition and processing, MPS are fully characterized by the process maps applied in each emission cycle to the auxiliary qubit and the respective photonic mode. To estimate the maximal size of states over which entanglement persists, we thus infer the entanglement length *D*_ent._ from measured process maps for the cluster and GHZ states, and from partial tomography for the W state as detailed in the following. We characterize the generated cluster and GHZ states for *N* > 4 considering the repetitive nature of the underlying photon emission processes. We tomographically measure the process map $\chi :{\rho }_{A}^{({\rm{pre}})}\to {\rho }_{A,P}^{({\rm{post}})}$ taking a generic input density matrix of the auxiliary qubit ${\rho }_{A}^{({\rm{pre}})}$ to its corresponding output state, while generating an entangled photonic qubit ${\rho }_{A,P}^{({\rm{post}})}$. This measured process map, which we assume to be the same for all *N* − 1 emission steps, except for the last one, allows us to infer the most likely density matrix within the class of matrix-product density operators with bond dimension *d* = 2^8^ (see Supplementary Note 5). We determine the negativity between the first and last photonic qubits from the density matrices calculated on the basis of the measured process maps (dashed lines), after projecting out all remaining qubits as before. We find a larger entanglement length *D*_ent.,GHZ_ ≃ 16 for the GHZ state, compared to the entanglement length *D*_ent.,Cluster_ ≃ 11 of the cluster state, in agreement with master equation simulations. The negativities obtained from measured process maps follow a similar trend as the ones obtained from master equation simulations, indicating that the experimental performance is mostly limited by decoherence of the auxiliary system, relative to the repetition time *T*. The slightly lower negativity for the process map approach is likely dominated by coherent errors in the gate operations.

For the W state, each emission step is a SWAP(*θ*_*i*_) gate with *θ*_*i*_ being different in each cycle *i*. Instead of measuring *N* different process maps, we opted for an alternative characterization method, by measuring the density matrices *ρ*_mix.,*i*_ between pairs of the first and the *i*-th photonic qubit. For an ideal *N*-qubit W state, we expect these density matrices to satisfy the relation ${\rho }_{i}=N/2\left[{\rho }_{{\rm{mix}}.,i}-(N-2)/N\left|00\right\rangle \left\langle 00\right|\right]$ with ${\rho }_{i}=\left|{{\rm{W}}}_{2}\right\rangle \left\langle {{\rm{W}}}_{2}\right|$ independent of *i*. The second term on the right originates from tracing out *N* − 2 photonic qubits. Motivated by these identities, we calculate the experimental *ρ*_*i*_ based on the measured *ρ*_mix.,*i*_ using the above relation for the W state of ten photonic modes. We estimate the degree of entanglement between the first and *i*-th photonic qubit from the negativity of *ρ*_*i*_ (empty dots). Also in this case, we find good agreement with the simulation results and an entanglement length on the order of ten.

## Discussion

As interesting future directions, one could explore applications of the presented source for one-way quantum computing with cluster states^6,26,27^, Heisenberg-limited metrology, or teleportation with GHZ states^28–30^, or photon loss resilient quantum communication with W states^31,32^. In addition, this versatile source of quantum many-body states of electromagnetic radiation, able to perform generic gates of the CNOT and SWAP families, could be used to access a larger variety of quantum many-body states in the MPS family, e.g., as ground states of variational quantum algorithms^33,34^. Finally, we note that our platform naturally allows for integration of additional auxiliary and emitter qubits, suggesting a path to explore entangled tensor network states in higher dimensions^35^.

## Supplementary information

Supplementary Information

## Data Availability

The authors declare that the data supporting the findings of this letter and corresponding Supplementary Information file are available online at the ETH Zurich repository for research data 10.3929/ethz-b-000429562.
