# Supplementary material for: Realizing a deterministic source of multipartite-entangled photonic qubits
Source: Nat Commun. 2020 Sep 28;11:4877. doi: 10.1038/s41467-020-18635-x (PMC7522291; doi:10.1038/s41467-020-18635-x)
Supplement: Supplementary file 1 — Supplementary Information [file 41467_2020_18635_MOESM1_ESM.pdf]

Supplementary Information
Realizing a Deterministic Source of Multipartite-Entangled Photonic Qubits

Jean-Claude Besse et al.

SUPPLEMENTARY NOTES

Supplementary Note 1: Fabrication and experimental setup

We fabricated the sample, shown in Fig. 2(a-d), on a 4.3 mm x 7 mm silicon substrate. We patterned all elements except for the Josephson junctions in a 150 nm-thick sputtered niobium film using photolithography and reactive ion etching. We fabricated the Josephson junctions in a separate step using electron-beam lithography and shadow-evaporation of aluminum in an electron-beam evaporator. We mounted the sample on the base temperature stage (20 mK) of a dilution refrigerator, inside an aluminum and a cryoperm shield, as shown in the wiring diagram in Supplementary Figure 1. We used a combination of coils, and flux lines equipped with low pass (LP, 780 MHz) and infrared filters based on Eccosorb CR-124 absorber material [1], for flux biasing of the sample. We attenuated the signals in the drive lines using a 20/20/20 dB scheme (at the 4 K, 100 mK, and base temperature stages) [2]. We operated both the readout output line, and the photon output with Josephson Parametric Amplifiers (JPA) [3] as first elements in the detection chains, with 27 dB and 18 dB of gain respectively. We placed them in separate sample holders with two layers of cryoperm shielding and a coil for magnetic flux biasing. At room temperature, we amplified the readout output (A) with one extra stage compared to the photon output (B). We made this choice to preserve the linearity in the amplification chain for the photonic modes, an essential requirement [4] to perform tomography. The use of an IQ combiner increases the efficiency of the detection chain when the gain of the JPA is not fully overcoming the noise added later in the chain.

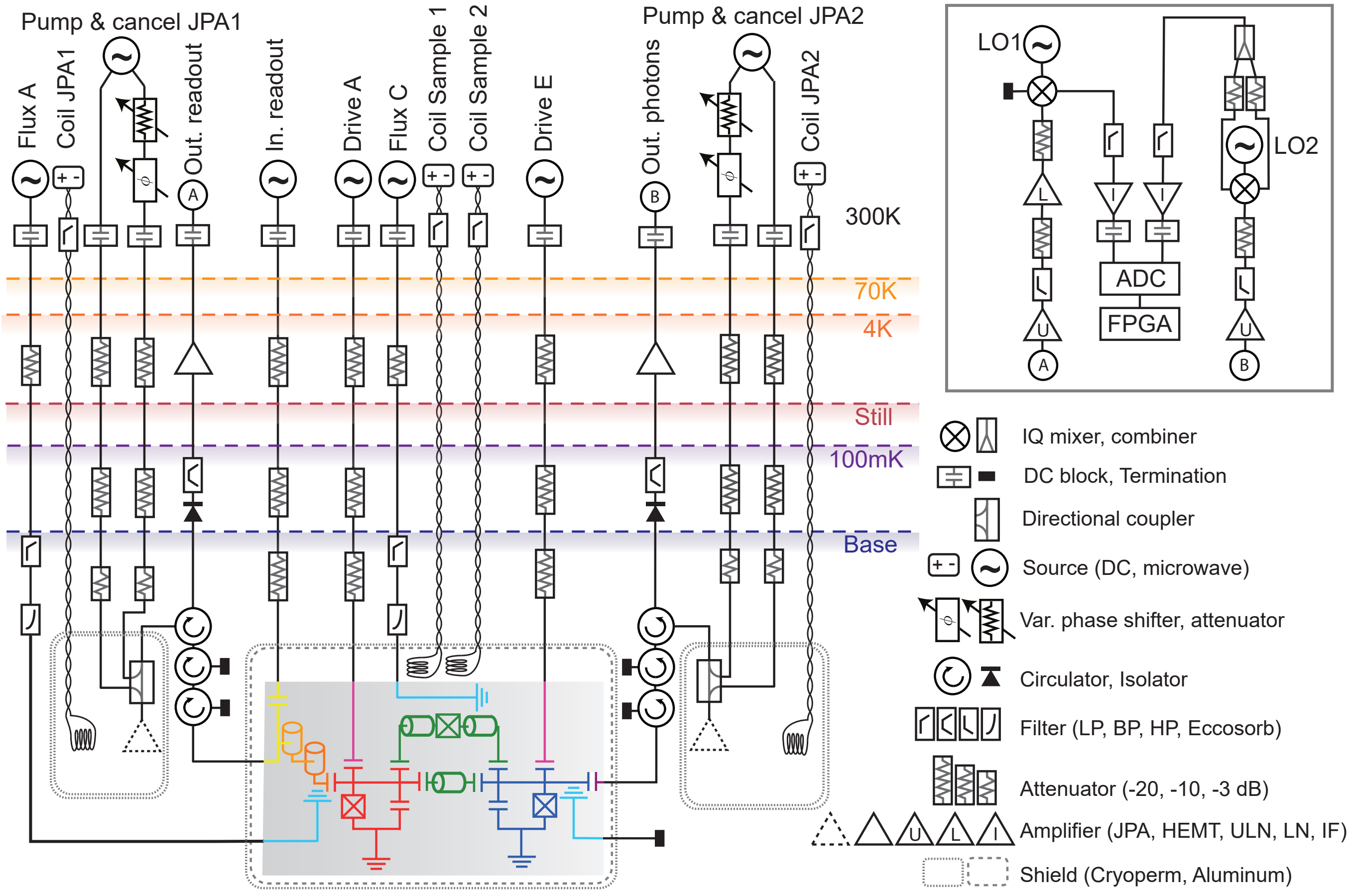

Supplementary Figure 1. **Schematic of experimental setup.** For details see main text.

Supplementary Note 2: Device characterization

We characterize first the auxiliary system using its dispersive coupling to the readout resonator and Purcell filter [5]. Transmission of a weak signal through the feedline shows the presence of a resonator mode at frequency $\omega_{\mathrm{ro.}}/2\pi = 6.664$ GHz, with effective linewidth $\kappa_{\mathrm{eff.}}/2\pi = 25$ MHz, when the auxiliary qubit is biased to its maximum frequency, the sweet spot (see Supplementary Table I for a summary of the parameters). We use qubit spectroscopy to find the transition frequencies $\omega_{ge}/2\pi = 5.758$ GHz and $\omega_{ef}/2\pi = 5.455$ GHz. Time-resolved resonator spectroscopy when preparing the first (second) excited state of the auxiliary qubit allows for the extraction of the dispersive shift of that state imparted on the readout resonator $\chi^{(e)}/2\pi = -5$ MHz ($\chi^{(f)}/2\pi = -8$ MHz).

In a second step, we use the coherent scattering of a weak input tone by the emitter qubit into the transmission line to determine its transition frequency. Using a bias coil, we tune it to $\omega_{01}/2\pi = 5.896$ GHz, such that both the SWAP frequency $\Delta_{\mathrm{e0g1}}/2\pi = 139$ MHz and the CNOT frequency $\Delta_{\mathrm{f0e1}}/2\pi = 441$ MHz are well separated from each other, far detuned from the anharmonicity $\alpha/2\pi = (\omega_{ef} - \omega_{ge})/2\pi = -303$ MHz, and within the bandwidth of the Arbitrary Waveform Generator (AWG) used to drive them.

We characterize the tunable coupler by driving it into a mixed state using a strong continuous drive on the charge line which weakly couples to it, and observing a dispersive shift on both the emitter and the auxiliary qubits. Tuned to $\omega_{\mathrm{c}}/2\pi = 4.7$ GHz, the tunable coupler imposes a coupling between the auxiliary and the emitter qubits that has the opposite sign as the coupling of the fixed coupler, such that the constant coupling J_{DC} vanishes. We verify experimentally that $J_{\mathrm{DC}}/2\pi < 20$ kHz is reachable by performing time-resolved T_1 and Ramsey sequences at smaller auxiliary-emitter detunings, and observe that the lifetime and Ramsey dephasing times of the auxiliary $|e\rangle$ and $|f\rangle$ states are not limited by

Auxiliary	g-e frequency, $\omega_{ge}/2\pi$	5.758 GHz
	e-f frequency, $\omega_{ef}/2\pi$	5.455 GHz
	anharmonicity, $\alpha/2\pi$	-303 MHz
	lifetime of $|e\rangle$, $T_1^{(e)}$	21 μs
	lifetime of $|f\rangle$, $T_1^{(f)}$	7 μs
	Ramsey dephasing time of $|e\rangle$, $T_2^{\star(e)}$	17 μs
	Ramsey dephasing time of $|f\rangle$, $T_2^{\star(f)}$	8 μs
	readout frequency, $\omega_{\mathrm{ro.}}/2\pi$	6.664 GHz
	readout linewidth, $\kappa_{\mathrm{eff.}}/2\pi$	25 MHz
	dispersive shift of $|e\rangle$, $\chi^{(e)}/2\pi$	-5 MHz
	dispersive shift of $|f\rangle$, $\chi^{(f)}/2\pi$	-8 MHz
Coupler	tunable coupler frequency, $\omega_{\mathrm{c}}/2\pi$	4.7 GHz
Emitter	0-1 frequency, $\omega_{01}/2\pi$	5.896 GHz
	decay rate, $\kappa/2\pi$	1.95 MHz

Supplementary Table I. Measured device parameters.

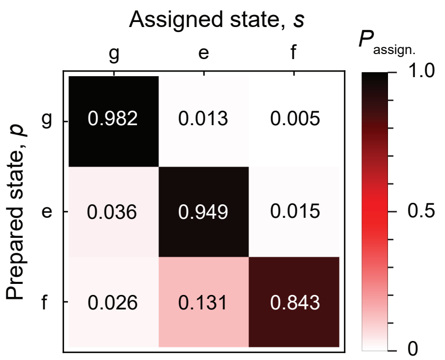

Supplementary Figure 2. **Qutrit readout.** Assignment probability matrix $P_{\mathrm{assign.}}(s|p)$ for the auxiliary system A prepared in state p, and assigned to state s.

their Purcell coupling to the emitter at the frequencies chosen for the experiment. We extract the strength of the parametrically activated couplings $J_{\mathrm{AC}}/2\pi \simeq 5$ MHz from the splitting of the emitter transition when parametrically tuned into resonance with a transition of the auxiliary qubit.

By comparing the Rabi-oscillation visibilities of the e-f transition with and without a $R_y^{ge}(\pi)$-pulse [6], we measure a steady-state thermally excited population of the auxiliary qubit of $n_{\mathrm{exc.}} = 0.08$. We realize an unconditional reset of the thermally excited population of the auxiliary qubit [7], by preceding all measurements reported in this manuscript by a 2 μs long pulse at the SWAP frequency Δ_{e0g1}. This equates the auxiliary system excited population to the steady-state population of the emitter qubit, which quickly thermalizes to the output transmission line thermal population through the engineered decay rate κ. After a reset pulse, we extract, in single-shot readout, as well as by comparing the Rabi-oscillations visibilities on the e-f transition with and without a $R_y^{ge}(\pi)$-pulse, an upper limit of $n_{\mathrm{th}} = 0.003$ of the excited-state population. This corresponds to a 40 mK equivalent temperature of the transmission line.

We perform three-level dispersive single-shot readout [7] in 256 ns with a fidelity characterized by the assignment probability matrix $P_{\mathrm{assign.}}$ presented in Supplementary Figure 2.

Supplementary Note 3: Two-qubit gates

We perform gates of the SWAP and CNOT families, which are represented with respect to the basis $B = \{|g0\rangle, |g1\rangle, |e0\rangle, |e1\rangle\}$ by an arbitrary rotation angle θ and phase ϕ as

$$\mathrm{SWAP}(\theta,\phi) = \begin{pmatrix} 1 & 0 & 0 & 0 \\ 0 & \cos\theta/2 & e^{i\phi}\sin\theta/2 & 0 \\ 0 & e^{-i\phi}\sin\theta/2 & \cos\theta/2 & 0 \\ 0 & 0 & 0 & 1 \end{pmatrix} \quad (1)$$

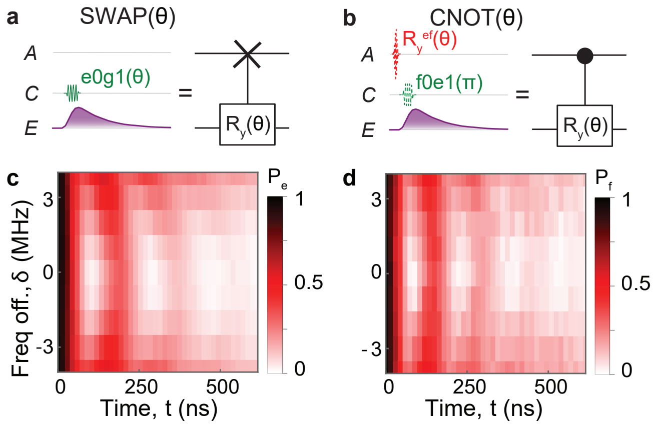

Supplementary Figure 3. **SWAP and CNOT gate calibration.** (a-b) Pulses applied to the auxiliary qubit (A) and the coupler (C) generating excitations in the emitter qubit (E), for a gate of the SWAP (a) or CNOT (b) family. (c)[(d)] Measured $|e\rangle$-state ($|f\rangle$-state) population as a function of pulse length t and pulse detuning δ from the sideband frequency Δ_{e0g1} (Δ_{f0e1}) for the pulse scheme shown in (a) [(b)].

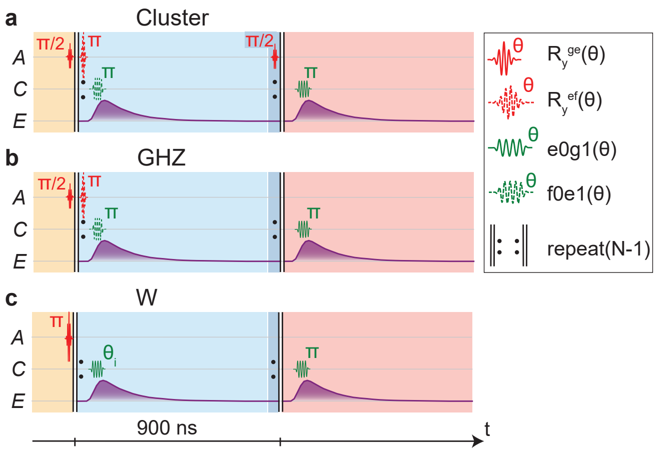

Supplementary Figure 4. **Emission sequences.** Pulses applied to the auxiliary qubit (A) and the coupler (C) generating excitations in the emitter qubit (E), in order to prepare (a) the cluster state, (b) the GHZ state, and (c) the W state. Time axis (horizontal) is to scale, with photons emitted every 900 ns.

and

$$\mathrm{CNOT}(\theta,\phi) = \begin{pmatrix} 1 & 0 & 0 & 0 \\ 0 & 1 & 0 & 0 \\ 0 & 0 & \cos\theta/2 & e^{i\phi}\sin\theta/2 \\ 0 & 0 & e^{-i\phi}\sin\theta/2 & \cos\theta/2 \end{pmatrix}. \quad (2)$$

In the work reported here, we always choose the phase $\phi = 0$ by an appropriate choice of the phase of the radio-frequency pulses, and start a two-qubit gate with the emitter in the state $|0\rangle$.

We perform SWAP-type gates by applying a radio-frequency pulse to the coupler flux line at the difference frequency $\Delta_{\mathrm{e0g1}}/2\pi = (\omega_{01} - \omega_{ge})/2\pi = 139$ MHz between the two states in the first manifold of excitations, Supplementary Figure 3(a). We calibrate the duration and exact frequency of this pulse by measuring the excited state population of the auxiliary qutrit after preparing the $|e0\rangle$ state, for a pulse frequency offset δ and a duration t, see Supplementary Figure 3(c). We adjust the duration of the pulse $t$and also its amplitude when high resolution is required to achieve a targeted rotation angle θ. We implement the CNOT-type gates in an analogous fashion: in a first step, we realize the conditional creation of an excitation by applying a $R_y^{\mathrm{ef}}(\theta)$-pulse on the second transition of the auxiliary qutrit. Then, we apply a microwave pulse to the coupler flux line at the difference frequency $\Delta_{\mathrm{f0e1}}/2\pi = (\omega_{01} - \omega_{ef})/2\pi = 441$ MHz between the two states in the second manifold of excitations, Supplementary Figure 3(b). We experimentally calibrate the duration and exact frequency of this pulse by preparing the $|f0\rangle$ state, and recording the population P_f remaining in the second-excited level of the auxiliary qubit for a pulse frequency offset δ and duration t, see Supplementary Figure 3(d). By always performing a pulse with the duration corresponding to a full transfer of excitation, and selecting the desired angle θ by the rotation angle of the excitation-creating pulse $R_y^{\mathrm{ef}}(\theta)$, we guarantee to have no population remaining in the non-computational state $|f\rangle$ after the gate.

We implement the protocol of Figure 1(a-b) through the application of microwave pulses to the auxiliary qubit charge line and the coupler flux line, according to the timing diagram shown in Supplementary Figure 4, where we plot the duration of the pulses to scale. We emitted all photons with the same time delay of $T = 900$ ns, chosen to be approximately one order of magnitude longer than the decay time of the emitter $\kappa^{-1} \simeq 80$ ns, such that the photonic time-bins are well separated. In this regime, the infidelity is dominated by incoherent errors caused by the relaxation of the auxiliary system during the time T. Further improvements in the fidelity and entanglement length could be achieved in the future by reducing the repetition time T and thereby finding the best trade-off between coherent errors, originating from time-bin overlap, and incoherent errors. A choice of larger decay rate κ of the emitter would also allow the repetition time to be decreased.

Supplementary Note 4: Complete photonic tomography

The procedure for complete tomography of photonic states with up to $N = 4$ modes is based on the $R\rho R$ method described in Ref. [4], and detailed below. The signal amplitude a_{out} emitted from the sample passes through an amplification chain whose first element is a JPA with 18 dB of phase-preserving gain. After further amplification and filtering, it is down-converted at room temperature to 250 MHz using a local oscillator, and

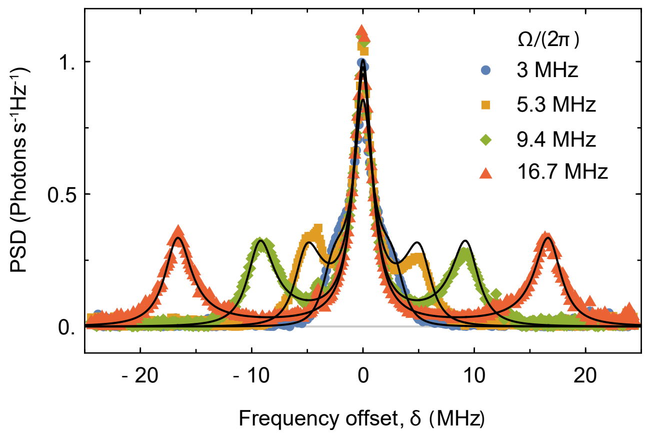

Supplementary Figure 5. **Absolute power calibration.** Measured power spectral density (PSD) of the inelastic scattering of a coherent tone resonant with the emitter qubit (symbols) for various drive rates Ω. Solid lines represent a global fit to the data.

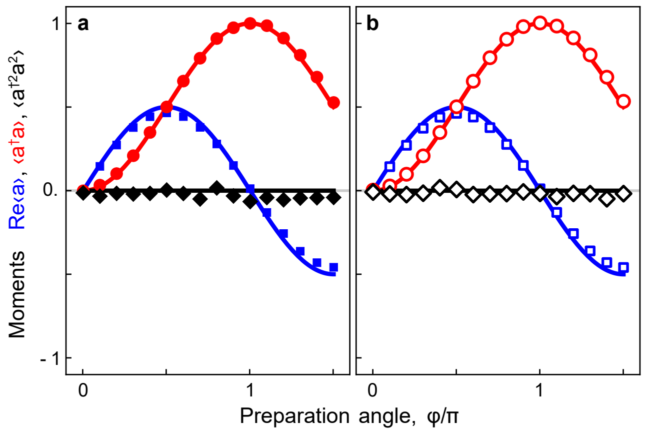

Supplementary Figure 6. **States in the single photon manifold.** Measured moments $\mathrm{Re}\langle a\rangle$ (blue squares), $\langle a^\dagger a\rangle$ (red circles), and $\langle (a^\dagger)^2 a^2\rangle$ (black diamonds), for superpositions $|\psi\rangle = \sin(\varphi/2)\,|0\rangle + \cos(\varphi/2)\,|1\rangle$ of vacuum and single photon states. The states are generated from the first manifold of the auxiliary qubit (a, filled markers) using SWAP gate, or the second manifold (b, empty markers) using a CNOT gate. Lines indicate ideal expectation values. Imaginary parts, not shown, are all below 0.05. Statistical error bars are smaller than the markers.

digitized at 1 GS/s by a analog-to-digital converter (ADC) passing the data on to a field-programmable gate array (FPGA).

We use the resonant photon-blockade of the emitter qubit under strong drive [8] to realize a calibrated power source and quantify the root-mean-square (RMS) voltage at the ADC input corresponding to one photon being emitted by our sample. This is performed by continuously, resonantly, driving the emitter qubit at rate $\Omega > \kappa$ and recording the inelastically emitted radiation, see Supplementary Figure 5. The nonlinear power spectral density (PSD), showing satellite peaks at detunings $\delta \simeq \pm\Omega$, and saturating to 1 photon s $^{-1}$ Hz^{-1}, is globally fitted as a Mollow triplet. This fit serves as a calibration for the emitted power $P = n_q \kappa \hbar \omega_{01}$. Here, $n_q \simeq 1/2$ is the steady-state average excited-state population of the emitter qubit under a large drive rate Ω.

On the FPGA, we integrate the amplified output signal $s_{i,\mathrm{out}}(t)$ in each time bin with a mode-matched filter $w_i(t)$ satisfying $\int |w_i(t)|^2\,\mathrm{d}t = 1$ to yield the complex amplitude $S_i = \int \mathrm{d}t\, w_i(t) s_{i,\mathrm{out}}(t) = I_i + iQ_i = a + h^\dagger$. Here, a is the mode of interest, and h the added noise in the detection chain. We collect a quadrature pair $\{I_i, Q_i\}$ per photonic mode P_i. We record two $2N$-dimensional histograms of the measured distributions of the quadratures, the first one (H_{on}) with the signal mode a in the emitted state $|\psi\rangle$ to be characterized, and added noise of the detection chain, the second one (H_{off}) with the signal mode a prepared in the vacuum state, i.e. recording noise added by the detection chain only. Memory constraints on the acquisition device limit the total number of histogram bins to 2^{24}, resulting in a resolution of $2^{24/2N}$ bins for each quadrature, which currently limits us to perform full tomography for up to $N = 4$ modes. Since the Mollow triplet based calibration described above provides us with an absolute scale for the histogram axes, we quantify the efficiency of the detection chain $\eta = (1 + n_{\mathrm{noise}})^{-1} \approx 0.29$, corresponding to $n_{\mathrm{noise}} \approx 2.5$ added noise photons, by computing the average photon number based in H_{off}. This efficiency is a bit lower than state of the art, due to the low gain (18 dB) chosen to preserve the linearity of the chain, such that the amplified vacuum noise by the JPA does not fully overcome the noise added later in the chain.

We verify the single photon character by measuring antibunching performing single mode tomography [9]. We prepare a quantum superposition of vacuum and a single photon in the Fock basis $|\psi\rangle = \sin(\varphi/2)\,|0\rangle + \cos(\varphi/2)\,|1\rangle$ in two different ways. The first is achieved by preparing the equivalent superposition between the ground and first excited state of the auxiliary qubit $|\psi_1\rangle = \sin(\varphi/2)\,|g\rangle + \cos(\varphi/2)\,|e\rangle$ and performing a SWAP gate. The second consists of the prepared superposition in the second manifold $|\psi_2\rangle = \sin(\varphi/2)\,|e\rangle + \cos(\varphi/2)\,|f\rangle$, followed by a $|f0\rangle - |e1\rangle$ transition. We evaluate the expectation values of the moments $\langle (a^\dagger)^n a^m\rangle$ up to $n, m < 3$ versus the preparation angle φ, see Supplementary Figure 6 for the real part of the amplitude $\mathrm{Re}\langle a\rangle$, the power $\langle a^\dagger a\rangle$, and the second-order correlator $\langle (a^\dagger)^2 a^2\rangle$. We verify that our gates lead to states in the single photon manifold, as the $\langle (a^\dagger)^2 a^2\rangle$ moments measured are all close to zero, with extreme values -0.07 and $+0.06$, corresponding to a vanishing second-order correlation function $g^{(2)}(0)$ at zero time delay. The emitted power $\langle a^\dagger a\rangle$ follows the expected $\sin^2(\varphi)$ dependence well in both cases. The coherence, characterized by the first order moment $\langle a\rangle$, is slightly reduced from the ideal value in both cases in agreement with the Ramsey-dephasing times of the $|e\rangle$ and $|f\rangle$ levels [by less than 1% in panel (a) and 2% in panel (b)].

For photonic states with multiple modes, we start by verifying that the second-order correlation function $\langle (a^\dagger)^2 a^2 \rangle$ vanishes in each individual mode. We reconstruct the density matrix of the noise mode ρ_h by defining positive operator-valued measures (POVMs) $\Pi_j = \Pi_{\otimes_i S_i} = \bigotimes_i \Pi_{S_i}$, where $\Pi_{S_i} = \pi^{-1} |\alpha = S_i\rangle \langle \alpha = S_i|$. We create one POVM per histogram bin, using the absolute scale found in the Mollow triplet fit Supplementary Figure 5. We then iteratively converge towards ρ_h by starting in a maximally mixed state $\rho_0 = \mathbb{1}/d$ and updating the estimate according to

$$\rho_{k+1} = NG^{-1} R(\rho_k) \rho_k R(\rho_k) G^{-1}, \tag{3}$$

where $R(\rho) = \sum_j H_{\text{off}}(j) \Pi_j [\text{Tr}(\rho \Pi_j)]^{-1}$, $G = \sum_j \Pi_j$, and N is a renormalization constant. This procedure guarantees convergence to the most likely physical density matrix ρ_h of the noise mode. Experimentally, we find very good agreement between the most likely matrix found via the iterative method and a thermal state $\rho_{\text{th.}} = \sum_{n=0}^{\infty} n_{\text{noise}}^n / (1 + n_{\text{noise}})^{(n+1)} |n\rangle \langle n|$ with $n_{\text{noise}} \approx 2.5$ noise photons. Nevertheless, we use the experimental noise mode ρ_h in the following. We then apply the iterative method a second time, with the knowledge of the noise mode enabling the creation of POVMs that are displaced noise modes $\Pi_{S_i} = \pi^{-1} D_{\alpha=S_i} \rho_h D^\dagger_{\alpha=S_i}$, with the displacement operator D, and using the histogram H_{on} instead of H_{off} to construct the update operator R.

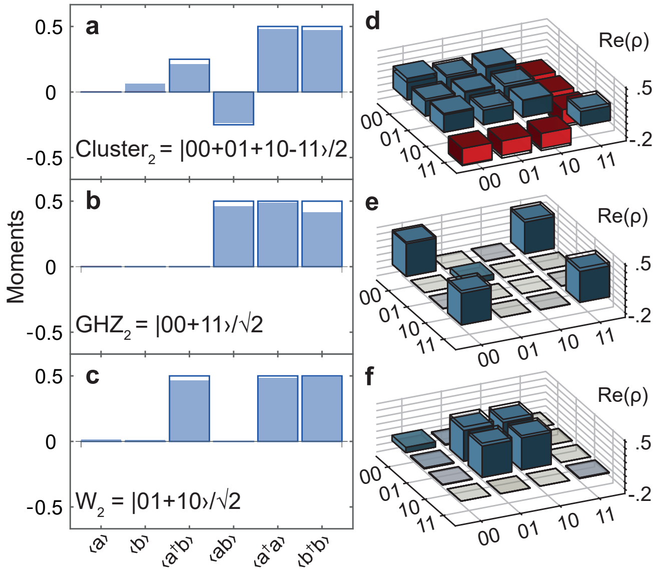

Supplementary Figure 7. **Two-mode states with Bell-type correlations.** Two-photon state tomography for (a,d) the cluster state, (b,e) the GHZ state (Bell state $|\Phi^+\rangle$) and (c,f) the W state (Bell state $|\Psi^+\rangle$). (a-c) Real part of the moments listed (blue bars), together with ideal values (solid blue wireframes). The operator a (b) refers to the first (second) time-bin defined photonic state. (d-f) Real part of the density matrices $\text{Re}(\rho)$ (bars) and ideal values $\text{Re}(\rho_{\text{ideal}})$ (black wireframes). All imaginary parts of moments and density matrix entries are below 0.02.

We reconstruct with this procedure the most likely density matrix ρ of the emitted state $|\psi\rangle$ under the physicality constraints, that is, the matrix is hermitian, positive semi-definite, and its trace is equal to 1.

We reconstruct the two-mode states, Bell states up to local rotations, emitted using the protocols described above, see Supplementary Figure 7. We observe, as expected, that each mode shows almost zero coherence when tracing out the other one (first order moments $\langle a \rangle$ or $\langle b \rangle$ close to zero). The cross-correlators of the form $\langle ab \rangle$ or $\langle a^\dagger b \rangle$ are non-zero, indicating entanglement between the two modes. We reconstruct density matrices with fidelities $F_{\text{Cluster}_2} = 0.94$, $F_{\text{GHZ}_2} = 0.92$, and $F_{\text{W}_2} = 0.91$, and negativities $\mathcal{N}_{\text{Cluster}_2} = 0.44$, $\mathcal{N}_{\text{GHZ}_2} = 0.42$, and $\mathcal{N}_{\text{W}_2} = 0.41$ (ideal value is 0.5) as a witness of entanglement.

Density matrices for the states with three and four photonic qubits are discussed in the main text.

Supplementary Note 5: Process maps

As motivated in the main text we estimate the density matrices for states with $N > 4$ within the class of matrix products density operators with bond dimension $d = 2$ by experimentally reconstructing the process map $\chi^{(l)}$ for individual emission processes and by calculating $\rho = (\Pi_{l=N}^{1} \chi^{(l)}) \rho_0$, where ρ_0 is the density matrix obtained after the initialization pulse in Figure 1. We further assume that the maps $\chi^{(l)}$ for nominally identical processes are independent of l, which assumes history-independent operations. This assumption is justified as we always perform the same operation, and after one photon is emitted, it does not interact with the auxiliary system anymore. In order to estimate ρ for the cluster and GHZ states we therefore need to characterize four process maps in total, for the CNOT, the Hadamard followed by a CNOT, the SWAP, and the Hadamard followed by a SWAP. Each process matrix $\chi^{(l)}$ maps an input state of the auxiliary qubit to an output state of the joint system consisting of the auxiliary qubit and the emitted photonic qubit $\chi : \rho_A^{(\text{pre})} \to \rho_{A,P}^{(\text{post})}$. In the Pauli basis, we write the auxiliary qubit input state $\rho_A^{(\text{pre})} = \sum_k \rho_k^{(\text{pre})} \sigma_k$ and the output joint system $\rho_{A,P}^{(\text{post})} = \sum_{i,j} \rho_{i,j}^{(\text{post})} \sigma_i \otimes \sigma_j = \sum_{i,j} \left(\sum_k \chi_{i,j}^k \rho_k^{(\text{pre})} \right) \sigma_i \otimes \sigma_j$.

We characterize a process map by recording correlations between the auxiliary system and the emitted photonic qubit. For each prepared cardinal state of the auxiliary qubit, $\{|g\rangle, |e\rangle, |g+e\rangle, |g-e\rangle, |g+ie\rangle, |g-ie\rangle\}$ (4 cardinal states would have been sufficient, but we use redundancy to reduce the impact of experimental errors), we record 3 state-conditioned 2D-histograms $H(I, Q, s)$ [10], where I and Q are mode-matched integrated quadratures of the photonic mode as in the previous section, and s is the state the auxiliary qutrit was assigned to, in single-shot dispersive readout [5], directly on the

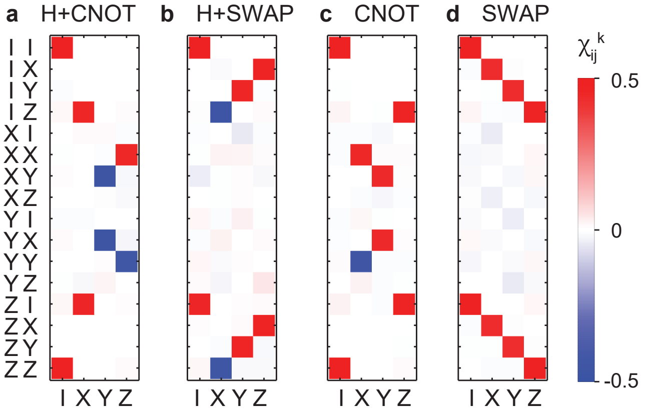

Supplementary Figure 8. **Measured process maps of repeatable operations.** Process maps χ in the Pauli basis. (a,b) Operations used for the cluster state preparation: H+CNOT (a), H+SWAP (b). (c,d) Operations used for the GHZ state preparation: CNOT (c), SWAP (d).

FPGA. The 3 histograms correspond to the X, Y, Z-bases of measurement for the auxiliary qubit. We correct for finite readout assignment fidelity by inverting the assignment probability matrix presented in Supplementary Figure 2, and by processing the histograms $\tilde{H}(I, Q, s) = \sum_{s'} P^{-1}_{\text{assign.}}(s'|s) H(I, Q, s')$.

We verify that the $|f\rangle$-state population is below 0.01 in all cases, allowing us to treat the auxiliary system as a qubit to very good approximation, and reconstruct the joint auxiliary and photonic qubits output density matrix for each of the six input states [10]. These matrices yield an overdetermined set of equations for the process map, of the form $\rho^{(\text{post})}_{i,j} = \sum_k \chi^k_{i,j} \rho^{(\text{pre})}_k$, which we solve for each fixed value of i and j using least squares, finding χ_{LS}. We impose physicality in a last step by ensuring that the map is trace preserving, and eigenvalues of the corresponding 8×8 Choi matrix C^χ are non-negative following Ref. [11]. We obtain the least squares Choi matrix $C^\chi_{\text{LS}} = \sum_{l,m} e_{l,m} \otimes \left(\sum_{i,j} \sum_k \chi^k_{i,j} \Lambda_{lm,k} \sigma_i \otimes \sigma_j \right)$ from the process map χ_{LS}. Here, $e_{l,m}$ is a 2×2-matrix with value 1 in the l, m-entry and 0 otherwise, and

$$\Lambda_{lm,k} = \frac{1}{2} \begin{pmatrix} 1 & 0 & 0 & 1 \\ 0 & 1 & i & 0 \\ 0 & 1 & -i & 0 \\ 1 & 0 & 0 & -1 \end{pmatrix} \tag{4}$$

is the transformation matrix from the Pauli basis to the computational basis. Choi's theorem guarantees that the process map is completely positive and trace preserving if and only if its Choi matrix is a positive matrix satisfying the conditions $\text{Tr}[C^\chi \sigma_0 \otimes \sigma_0 \otimes \sigma_0] = 2$ and $\text{Tr}[C^\chi \sigma_i \otimes \sigma_0 \otimes \sigma_0] = 0 \; \forall i \neq 0$. We find numerically the matrix C^χ which minimizes the distance to C^χ_{LS} (measured as the norm of the difference of the two matrices) under these constraints of physicality. The corresponding process maps, shown in Supplementary Figure 8, have real entries in a Pauli transfer matrix representation, and are close to the ideally expected ones with fidelities $F_{\text{CNOT}} = 0.924$, $F_{\text{SWAP}} = 0.918$, $F_{\text{H+CNOT}} = 0.923$, and $F_{\text{H+SWAP}} = 0.928$. The fidelity is defined as $F = \text{Tr}\left(\sqrt{\sqrt{C^\chi} C^\chi_{\text{ideal}} \sqrt{C^\chi}}\right)^2$, by comparing the experimental Choi matrix C^χ to the ideal one C^χ_{ideal}. We note that the H+SWAP and SWAP processes do not perform at the coherence limit, likely because of a sub-optimal calibration of the frequency and duration of the $e0 - g1$ pulse for this particular dataset. The fidelities could be improved in future experiments by using finer steps in the calibration procedure.

SUPPLEMENTARY REFERENCES

[1] D F Santavicca and D E Prober, "Impedance-matched low-pass stripline filters," Meas. Sci. Technol. **19**, 087001 (2008).

[2] S. Krinner, S. Storz, P. Kurpiers, P. Magnard, J. Heinsoo, R. Keller, J. Lütolf, C. Eichler, and A. Wallraff, "Engineering cryogenic setups for 100-qubit scale superconducting circuit systems," EPJ Quantum Technology **6**, 2 (2019).

[3] C. Eichler, Y. Salathe, J. Mlynek, S. Schmidt, and A. Wallraff, "Quantum-limited amplification and entanglement in coupled nonlinear resonators," Phys. Rev. Lett. **113**, 110502 (2014).

[4] C. Eichler, D. Bozyigit, and A. Wallraff, "Characterizing quantum microwave radiation and its entanglement with superconducting qubits using linear detectors," Phys. Rev. A **86**, 032106 (2012).

[5] T. Walter, P. Kurpiers, S. Gasparinetti, P. Magnard, A. Potočnik, Y. Salathé, M. Pechal, M. Mondal, M. Oppliger, C. Eichler, and A. Wallraff, "Rapid, high-fidelity, single-shot dispersive readout of superconducting qubits," Phys. Rev. Applied **7**, 054020 (2017).

[6] X. Y. Jin, A. Kamal, A. P. Sears, T. Gudmundsen, D. Hover, J. Miloshi, R. Slattery, F. Yan, J. Yoder, T. P. Orlando, S. Gustavsson, and W. D. Oliver, "Thermal and residual excited-state population in a 3d transmon qubit," Phys. Rev. Lett. **114**, 240501 (2015).

[7] P. Magnard, P. Kurpiers, B. Royer, T. Walter, J.-C. Besse, S. Gasparinetti, M. Pechal, J. Heinsoo, S. Storz, A. Blais, and A. Wallraff, "Fast and unconditional all-microwave reset of a superconducting qubit," Phys. Rev. Lett. **121**, 060502 (2018).

[8] C. Lang, D. Bozyigit, C. Eichler, L. Steffen, J. M. Fink, A. A. Abdumalikov Jr., M. Baur, S. Filipp, M. P. da Silva, A. Blais, and A. Wallraff, "Observation of resonant photon blockade at microwave frequencies using correlation function measurements," Phys. Rev. Lett. **106**, 243601 (2011).

[9] C. Eichler, D. Bozyigit, C. Lang, L. Steffen, J. Fink, and A. Wallraff, "Experimental state tomography of itinerant single microwave photons," Phys. Rev. Lett. **106**, 220503 (2011).

[10] C. Eichler, C. Lang, J. M. Fink, J. Govenius, S. Filipp, and A. Wallraff, "Observation of entanglement between itinerant microwave photons and a superconducting qubit," Phys. Rev. Lett. **109**, 240501 (2012).

[11] I. Schwartz, D. Cogan, E. R. Schmidgall, Y. Don, L. Gantz, O. Kenneth, N. H. Lindner, and D. Gershoni, "Deterministic generation of a cluster state of entangled photons," Science **354**, 434–437 (2016).
